# Targeting of Oral Bacterial Taxa Linked to Disease by Flavonols

**DOI:** 10.3390/nu18193123

**Published:** 2026-09-23

**Authors:** Michaela Tamamidis, Anthony Lopez, Wei Li, Stefan J. Green, Christine Wu, Guy R. Adami

**Affiliations:** 1 Department of Oral Medicine and Diagnostic Sciences, College of Dentistry, University of Illinois Chicago, 801 S. Paulina St., Chicago, IL 60612, USA; mtama4@uic.edu (M.T.); alope249@uic.edu (A.L.); 2 Department of Pediatric Dentistry, College of Dentistry, University of Illinois Chicago, 801 S. Paulina St., Chicago, IL 60612, USA; liw72@uic.edu (W.L.); chriswu@uic.edu (C.W.); 3 Genomics and Microbiome Core Facility, Rush University, Chicago, IL 60612, USA; stefan_green@rush.edu

**Keywords:** flavonol, ex vivo, oral biofilm, 16S rRNA, kaempferol, quercetin, myricetin, cardiovascular disease

## Abstract

Background: Networks of different bacterial taxa contribute to oral health. Antibacterials ideally would selectively modulate the subset of bacteria contributing to disease. Plant-derived natural compounds have been shown to inhibit planktonic bacteria or monospecies oral biofilms. However, very few in vivo studies are available to demonstrate the effects of these antimicrobials against native biofilms found in the human oral cavity. The objective of this study was to test putative antibacterials for their ability to affect specific bacterial taxa in complex biofilms. Methods: Oral biofilms from donors were collected from saliva then rapidly prepared for polyphenol testing. They were then incubated with test polyphenols for 4 h. 16S rRNA was measured using NGS cDNA sequencing to determine potential targets of bacterial inhibition. Results: Two flavonols, kaempferol and quercetin, had small inhibitory effects on total RNA levels while myricetin at the higher concentration tested, 1 mM, showed substantial inhibition. Effects on the overall microbial population structure as evidenced by alpha diversity of the oral biofilm 16S rRNA were minimal. Remarkably, the flavonols specifically lowered rRNA levels of oral bacterial species associated with both periodontal disease and cardiovascular disease while sparing many commensal bacteria taxa. Conclusions: In sum, plant-derived flavonols are a potential source of selective antimicrobial compounds against oral pathogens and bacteria that may contribute to systemic disease with minimal disruption of overall bacterial activity levels.

## 1. Introduction

### 1.1. Oral Bacteria Networks and Human Health

The oral microbiome is one of the largest and most complex host symbiosis environments in the human body. Home to over seven hundred taxa [1], it is a dynamic ecosystem composed of distinct microbial communities [2]. Rather than existing in isolation, these bacteria form interconnected networks through multispecies biofilms that allow for adhesion to tooth and epithelial surfaces. Within these biofilms, synergistic interspecies interactions such as nutritional cross-feeding as well as antagonistic behaviors like competition shape the microbial ecosystem. At the same time, these microbial networks constantly face environmental stressors from diet, saliva, oral hygiene practices, chewing, and competition from exogenous pathogens [3]. Thus, oral health is dependent on the homeostatic balance between the resident microbial communities and the host [1,4,5]. Disruptions in these bacterial networks can result in dysbiosis, leading to oral diseases like periodontitis, caries, gingivitis, and cancer.

The state of the oral microbiome may play a role in systemic health, affecting the microorganismal makeup of the brain and gut through the oral–brain and oral–intestinal axes respectively [6,7]. Oral bacteria presence in the gut [7,8] may lead to a number of systemic illnesses including gastrointestinal diseases such as inflammatory bowel disease, liver cirrhosis, and colon cancer. Oral bacteria or their products can translocate to distal organs in the case of cardiovascular diseases such as atherosclerosis [9] and neurodegenerative diseases like Alzheimer’s disease [10]. For those reasons, in theory, shifts in these complex oral bacterial networks may cause systemic disease.

### 1.2. Polyphenols and Bacterial Targeting

Flavonols are a member of the polyphenol family [11,12,13] with high abundance in fruits and vegetables characterized by a hydroxyl group on the C3 carbon of the phenolic ring with varying methylation and hydroxylation patterns [14,15,16]. Apart from their antioxidant properties, flavonols also have antimicrobial properties against bacteria, a role they serve in plants. Proposed mechanisms include targeting bacterial cell membrane integrity through hydrogen peroxide production, disrupting nucleic acid synthesis by inhibiting topoisomerase, and limiting energy metabolism via ATP synthase inhibition [17].

Numerous in vitro studies have tested polyphenol antimicrobial properties against gut bacteria typically using monoculture but ideally mixed culture methods. A study conducted by Whitman et al. found that polyphenol addition to an in vitro stool fermentation model led to increases in key gut taxa involved in metabolizing nutrients, including species of the genera *Bifidobacterium*, *Lactobacillus,* and *Ruminococcus bromii* [17,18]. Another in vitro study found enrichment in fecal species involved in maintaining gut health such as *Anaerostipes hadrus*, *Faecalibacterium prausnitzii*, and *Ruminococcus bromii* following targeting from blueberry-derived polyphenol fractions [18,19]. These studies and others show that flavonols or their metabolites can damage certain bacteria in various ways while sparing others [20,21,22,23].

Flavonols such as myricetin, quercetin, and kaempferol have also been shown to exert antimicrobial effects against bacteria involved in periodontal disease and caries, however most studies are centered on gut microbiota. An in vitro study focused on *Porphyromonas gingivalis*, a keystone periodontal disease pathogen, found a significant decrease following incubation in liquid culture with myricetin, with minimal inhibitory concentration (MIC) values between 62.5–125 μg/mL and minimal bactericidal concentration (MBC) values of 125–250 μg/mL [24]. Another in vitro study with myricetin and kaempferol isolated from crude methanol clove extract reported significant inhibition in the growth of periodontal pathogens *P. gingivalis* and *P. intermedia*, with an MIC of 20 μg/mL for each with both flavonols [25]. Similar effects have also been reported for quercetin. He et al. reported on quercetin’s ability to disrupt *P. gingivalis* biofilm formation using a broth microdilution assay of planktonic cultures, with MIC and MBC values of 200 μM and 400 μM respectively [26]. In a study conducted by Patra et al., quercetin and kaempferol were isolated from crude extracts and fractions from *Phytolacca americana*, and demonstrated inhibition in the growth of monocultures of oral pathogen *Porphyromonas gingivalis* and to lesser degree *Streptococcus mutans* [27]. Overall, these studies suggest flavonols exhibit antimicrobial effects in vitro and may hold potential as adjunctive therapeutic strategies to treat and prevent oral diseases such as periodontitis and dental caries.

### 1.3. Dietary Flavonol Intake and Systemic Health

Flavonol intake is thought to offer numerous health benefits including antioxidant, antihypertensive, and anti-allergic effects [11]. A meta-analysis of eight studies found a significant inverse association between high flavonol intake and stroke risk, with an increase of 20 mg/day of flavonol intake being associated with a 14% decrease in stroke risk [28]. Another meta-analysis of prospective cohort studies found that a 25 mg/day incremental increase of flavonol intake is associated with a 5% reduction in coronary heart disease risk [29]. In addition to cardiovascular benefits, dietary flavonol intake is proposed to offer anti-inflammatory and neurological benefits. A cross-sectional analysis of 2375 participants from the Framingham Heart Study Offspring Cohort found that higher flavonol intake was associated with decreased levels of cytokines and oxidative stress biomarkers, suggesting that these anti-inflammatory effects may play a role in reducing chronic disease risk [30]. In terms of neurological benefits, a large biracial study found higher dietary intake of the flavonols myricetin, kaempferol, and quercetin to have a protective function against cognitive decline as assessed by various cognitive performance tests [31].

Flavonols like myricetin, quercetin, and kaempferol are mainly found in fruits and vegetables such as cherries, tomatoes, blueberries, apricots, apples, grapes, leeks, ginger, broccoli, red cabbage, yellow onion as well as teas [11,32]. Table 1 summarizes the concentrations of these three flavonols in everyday fruits and vegetables based on the 2018 USDA Database for Flavonoid Content.

The hypothesis is that polyphenols modulate a subset of taxa of the oral bacterial network and that on balance the effect will be compatible with good health. Earlier work suggested some differences in the properties of the three related flavonols which differ based on the number of phenol moieties on the molecule though they are not typically tested side by side [17,33,34]. The flavonols can be tested using the shed saliva biofilm ex vivo assay which defines specific bacteria taxa affected by the tested agents in a microenvironment thought to mimic that of the oral cavity [35]. This assay takes advantage of the fact that oral biofilm is shed in large aggregates into saliva [36,37,38]. These aggregates of bacteria cycle on and off oral surfaces and may better mimic intact biofilm in vivo than artificial systems.

## 2. Materials and Methods

### 2.1. Sample Collection, Preparation and Exposure to Flavonols

Shed oral biofilm was collected from 10 participants with good overall health who had not taken antibiotics for the past 3 months, did not use tobacco and lacked oral soft tissue lesions. Verbal and written informed consent were obtained by all participants prior to saliva collection. All experiments were performed in accordance with relevant human participant research guidelines and regulations. Twenty-four hours before supplying the saliva samples, the participants were directed to refrain from oral care. Samples were collected between 9:00 a.m. and 12:00 p.m., where participants chewed 1 g of flavorless gum base for 5 min to stimulate saliva production. Following saliva collection, the samples were put on ice, centrifuged for 5 min at 4 °C at 5500× *g*, washed with transport medium, resuspended in PBS, and adjusted to OD_600nm_ = 1.0. As described earlier, shed biofilm in 400 μL of modified, supplemented SHI medium plus 100 μL of test agent was incubated at 37 °C for 4 h in an anaerobic chamber (Forma, Waltham, MA, USA) with anaerobic gas (5% CO_2_, 10% H_2_, 85% N_2_) [39]. All test samples had a 1.0% DMSO end concentration, including controls with no agent (contained water). The same process was repeated in the validation study with 6 additional participants. Quercetin (95% HPLC grade, CAS 117-39-5, Q4951) was obtained from Millipore Sigma (Burlington, MA, USA), while Myricetin (>98% purity, CAS529-44-2, 10012600) and Kaempferol (>98% purity, CAS 520-18-3, 11852) were obtained from Cayman Chemical (Ann Arbor, MI, USA) and stored as 100 mM stocks in DMSO stored at −80 °C and diluted 1/100 or 1/250 in test media.

### 2.2. RNA Extraction and Sequencing

Following incubation in anaerobic conditions, 2 volumes of RNA Protect Cell (Qiagen, Tokyo, Japan) were added to every test tube to preserve nucleic acid composition and stop any reactions. Samples were then incubated on ice for 30 min, followed by centrifugation at 5550× *g* for 10 min and removal of supernatant prior to storage at −80 °C. RNA was extracted using acid phenol followed by exposure to 1 unit/mL Turbo DNase for 30 min at 37 °C followed by silica-based purification with the Zymo Research RNA Clean and Concentrator-5 kit as described earlier [35]. From the original bacterial pellets, 1% of each harvested sample was used for cDNA synthesis with random sequence hexamers and Superscript III (Invitrogen, Thermo Fisher Scientific, Waltham, MA, USA). RNA measurement was based on concentration of total RNA isolated dissolved in 100 μL H_2_O and was by A260 absorption. For a number of samples a Qubit (Thermo, Waltham, MA, USA) and the Accu Green High Sensitivity DS quantitation Solution (Biotium, Fremont, CA, USA) were used to verify DNA was at levels that were undetectable. The V1–V3 variable region of bacterial 16S rRNA genes was amplified with 27F primer: CTACACGACGCTCTTCCGATCTAGRGTTYGATYMTGGCTCAG and 534R primer: CAGACGTGTGCTCTTCCGATCTATTACCGCGGCTGCTGG using KAPA HiFi HotStart PCR A second PCR amplification was conducted at the Rush University Genomics and Microbiome Core Facility where unique dual indexing sample-specific barcodes were added to each sample followed by cleanup and quantitation using the Illumina MiniSeq sequencer with a mid-output flow cell [40]. Final sequencing was performed on the Illumina MiSeq using a MiSeq Reagent Kit v3 (600-cycle), generating paired-end reads of 2x300 bp (309 nt in each direction) (Illumina, Inc, San Diego, CA, USA) at the DNA Services Lab, Roy J. Carver Biotechnology Center, at the University of Illinois at Urbana-Champaign. Negative controls contained water instead of saliva DNA and any additional controls included technical replicates of selected assays.

### 2.3. Microbial Community Analysis

16S rRNA amplicon FASTQ files were analyzed using the software package QIIME2 (v 2026.4) for taxa assignment and measurement. The sequences prior to merging had an average quality score of 35 and a size of 309 bases. To denoise the sequences after primer trimming, the DADA2 QIIME2-plugin was used to generate feature data and tables on the paired DNA sequence dataset. The Blast + consensus taxonomy classifier was used to assign taxonomy and determine query sequences with ≥98% match identity to the Human Oral Microbiome Database (v16.03). The cDNA amplicon read number ranged from 37,560 to 76,458 for the samples, with an average of 50,600 reads per sample.

MicrobiomeAnalyst was used to assess alpha and beta diversity on the feature level. PERMANOVA was used to assess the contribution of sample donor and treatment exposure to the derived beta diversity (Bray–Curtis Distances) across all samples and PERMDISP to test of homogeneity of multivariate dispersion when comparing groups.

Statistical analysis was conducted using R Studio (version 4.5.1). Differential abundance of bacterial RNA with and without the polyphenols tested were analyzed using Multivariate Association with Linear Models (MaAsLin3 version 1.2.0) using the default settings with Total Sum Scaling normalization of taxa read counts followed by log transformation [41]. Using MaAsLin3’s median-comparison feature abundance coefficients were tested against the median coefficient across taxa. The fixed effect was treatment type/concentration and the random effect was the donor ID. *p* Values were corrected for multiple testing using Benjamini–Hochberg method and a False Discovery Rate (FDR) of 0.1 or 0.05. Bacterial analysis was conducted on the species level, with a minimum abundance of 0.0001 and a minimum prevalence of 0.30. MaAsLin3 absolute effect sizes were compared among the three flavonols tested for the same bacterial taxa. Differences were assessed using a Friedman rank-sum test followed by a post hoc paired Wilcoxon signed-rank test with Benjamini–Hochberg correction. Both vehicle controls for each sample were included in the alpha diversity and RNA concentration analysis as a baseline. Bulk RNA concentrations after exposure to each agent were compared to controls and the significance of differences determined using a paired Student’s *t*-test. Frequencies and percentages were used to summarize categorical variables and mean ± standard deviation was used to present continuous variables.

## 3. Results

Human saliva was used as the source for shed oral biofilm because it is a reservoir of microorganism biofilm from all ecological niches in the human oral cavity with long-term stability. Stimulated saliva was obtained from a group of 10 people between 9:00 a.m. and 12:00 p.m., with volumes ranging from 10 to 28 mL. Donors had refrained from oral care over the previous 24 h and ranged in age from 20 to 67 years, with four females. Each sample was rapidly separated from the liquid phase, washed, and aliquoted for testing and placed in an anaerobic chamber where it was exposed to 1 of 3 flavonols and vehicle.

Flavonol concentrations were 400 μM and 1 mM. Even before eating cumulative saliva polyphenol concentrations are several millimolar [42]. While levels of individual flavonols are quite high in some foods, concentrations are limited by solubility both in vivo and ex vivo experiments [43,44,45]. While 400 μM saliva concentrations of quercetin have been documented after meals [45], the higher 1 mM concentration was chosen as it is achievable in theory based on dietary intake, though it is unclear how the flavonols are distributed between the aqueous and biofilm components in vivo or in these ex vivo experiments. After addition of each flavonol or carrier, shed biofilm samples were incubated anaerobically for 4 h at 37 °C in test medium. RNA was then isolated, subjected to cDNA synthesis and measurement of the 16S rRNA V1–V3 region using next generation DNA sequencing (NGS). All analyses were paired during the linear regression analysis with a comparison of the 16S rRNA profile in the mock-treated shed biofilm versus that of the samples exposed to the various flavonols. FDR < 0.1 was used to identify potential taxa with different rRNA levels for the 10-sample exploratory group. The heat map in Figure 1 reveals taxa rRNA that were determined to be at high or lower relative levels after the exposure to the different flavonols. Many taxa were affected by two or three of the flavonols tested.

Figure 2 shows myricetin had the largest mean and median absolute effect sizes at 400 μM and 1 mM. A Friedman rank-sum test at 400 μM and 1 mM showed significant differences in effect size magnitudes among the three flavonols. A post hoc paired Wilcoxon signed-rank test with at 400 μM and 1 mM showed significant differences between myricetin and kaempferol (400 μM FDR = 0.353 × 10^−3^, 1 mM FDR = 1.19 × 10^−11^) as well as myricetin and quercetin (400 μM FDR = 1.30 × 10^−2^, 1 mM FDR = 1.67 × 10^−13^) using Benjamini–Hochberg correction.

To validate identification of these target taxa, a similar analysis was done with shed biofilm with six additional human donors, age 19 to 35 years with four females, at FDR < 0.05. In sum, the direction of change versus the control needed to be the same and the difference over the six individuals had to be significant at FDR < 0.05 for validation. Many of the same taxa were altered in relative 16S rRNA levels in both trials by at least one flavonol (Figure 3). Figure 3 and Supplemental Table S1A,B reveal the taxa which are altered in relative 16S rRNA levels in both trials by at least one flavonol.

An important question was what effects the flavonols have on alpha diversity of the rRNA of the bacterial population overall. After calculation of the alpha diversity measure, a paired *t*-test was used to determine differences between flavonol and mock-treated shed biofilm. For this analysis both sets of samples were examined at once to increase N. Only myricetin at 1 mM had an effect on richness—actually showing an increase in Chao1 values (*p* < 0.00468 and FDR < 0.0281) (Figure 4A,B). For other tests, FDR values were, K0.4, FDR < 0.177; K1.0, FDR < 0.197; M0.4 FDR < 0.178; Q4 FDR < 0.197; Q1.0 FDR < 0.178. Differences in Shannon diversity, which is a measure of both species richness and evenness of spread, were recorded after correction for multiple testing for myricetin at 0.4 mM at *p* < 0.0027 and FDR < 0.0160. All other values were: K0.4, FDR < 0.94; K1.0, FDR < 0.78; M1.0 FDR < 0.459, Q 4 FDR < 0.174, Q1.0 FDR < 0.974.

Beta diversity differences with flavonol exposure are revealed in Figure 4C–F, which shows that the most active flavonols at the higher concentration had some effect on taxa levels in the populations studied based on PERMANOVA analysis. An examination of total RNA levels in shed biofilm samples after 4 h incubation with the various flavonols showed mid to high decreases in levels compared to the negative controls incubated with vehicle (Figure 4G).

## 4. Discussion

This work has demonstrated oral bacteria whose rRNA levels are targeted by three flavonols. While rRNA production is generally representative of bacterial activity, that is not always the case [46]. All rRNA levels measured are relative to the total in each sample which is in turn compared to the negative control for that person. Since bulk RNA levels were noted to stay the same or slightly decline for the different treatments versus the control, deficits in specific rRNA levels versus the controls incubated with vehicle are true deficits, but by no means indicate loss of bacteria. For taxa whose 16S rRNA were enriched with treatment, the results may be due to bacteria proliferation, increased buildup of RNA per cell or lack of inhibition to either.

The three flavonols tested vary in the number of hydroxyl groups but are otherwise quite similar. That may or may not explain why the bacteria targeted to show lower levels of 16S rRNA were largely similar for the three flavonols tested. Most notable is that many of the targets of the flavonols tested were potential oral pathogens, thus supporting the positive role of polyphenol consumption in health. One might speculate on a mechanism of cardiovascular effects. For example, of 17 oral taxa widely associated with the cardiovascular disease atherosclerosis [47,48,49,50,51], based on localization within sites of disease, and/or association of oral levels with the disease, seven taxa were prevalent in enough donors to be tested. Of these seven, four were shown to be inhibited by one or more of the flavonols (Figure 5). None showed increases in relative 16S rRNA levels.

The flavonols also favored inhibition of rRNA levels in bacteria involved in oral disease. The following taxa associated with periodontal disease severity were inhibited, *Parvimonas micra* [52], *Streptococcus anginosus* and *Streptococcus intermedius* [53,54,55], the latter two most firmly linked to periodontal abscesses. It is important to note that donors in this study largely lacked periodontal disease so only some periodontal pathogens were present in sufficient number of donors to be tested. Also, out of bacterial taxa rRNA inhibited in both datasets tested (Figure 3 and Supplement Table S1A,B) of the taxa were associated with caries. That included: *S. anginosus* and S. *intermedius* [56]. In contrast, none of the group of six bacteria taxa validated as enriched versus controls in the presence of flavonols was associated with oral disease [57]. Several of the taxa stimulated on the rRNA level are thought to be beneficial bacteria strongly associated with health such as *Streptococcus salivarius, Neisseria mucosa* and *Lautropia mirabilis* along with *Streptococcus infantis* [58,59]. Examination of other features to differentiate taxa rRNA inhibited versus that not inhibited by the three flavonols show fewer differences. Some taxa are found on teeth and some are on mucosa. Of those inhibited, 12 out of 13 were gram positives, while of those unaffected four out of six were gram positive. Curiously, bacteria with lower levels of rRNA were overwhelmingly anaerobes, with three obligate anaerobes and 10 facultative anaerobes, while bacteria whose relative rRNA expression was enriched in the presence of added flavonol included five facultative anaerobes and two aerobes, *N. mucosa* and *L. mirabilis*.

Myricetin was shown to affect relative rRNA levels of the most taxa in this study. One can speculate about reasons for that. The most obvious explanation would be higher reactivity. A recent study showed myricetin, which has the most phenolic hydroxyl groups of the 3, was also the most active against intracellular *Staphylococcus aureus* versus quercetin and kaempferol [60]. Second, higher solubility can also contribute to higher activity. With most polyphenols aqueous solubility can be limited, which is true for all three flavonols tested here [61]. While stock solutions of each flavonol tested were fully dissolved in DMSO it is hard to know what the actual concentration is in the test media plus biofilm after dilution. Third, compound stability can play a large role in activity of an agent. It has been reported that saliva contributes to polyphenol turnover. The evidence indicates that bacterial, not host microbial enzymes, in saliva largely play this role [62]. It is likely these enzymes would be well represented in the bacterial biofilm samples used. These same flavonols from additional sources will need to be tested to verify the high level of activity of myricetin.

A study with more people and different concentrations of the three flavonols tested will need to be done to determine just how similar or different effects on oral biofilm taxa are. It should be noted that the assay measured 16S rRNA levels and so is more an indicator of activity not viability [46,63]. Interestingly earlier work showed that another flavonoid had inhibitory, but not lethal, effects on many of the early oral colonizer taxa similarly identified here as inhibited [64]. Only one treatment caused major shutdown of bacterial rRNA levels—1 mM Myricetin (Figure 4). Surprisingly, despite deficient total RNA versus controls, this resulted in little decrease in alpha diversity, suggesting that mainly the more common taxa were affected. The other flavonols, and myricetin at lower concentration, had reproducible but lesser effects on total RNA levels showing 12 to 30% deficits versus control over the 4 h exposure. Somewhat unexpectedly, effects on alpha diversity were minimal. These were tested in paired analysis to decrease effects of differences in donor baseline oral microbe populations.

There are three main ways an antibacterial can directly inhibit activity of oral bacteria. One is to alter components of the bacteria so it can no longer proliferate. A second is to inhibit important processes in general, leading to cell death. A third is to alter adhesion properties of the bacteria so it is no longer part of the biofilm. While in theory the first two can be monitored in the shed biofilm assay by measuring rRNA synthesis, it is not clear how loss of adhesion would affect transcriptional activity—it is likely it would change but overall activity could either go up or down. For that reason, measuring 16S rRNA levels might not be as informative as measuring mRNA to determine antibacterial effects. The mechanism of bacterial activity inhibition by the flavonols remains to be uncovered.

A deficit in the work is the long incubation time used to determine flavonol-effected bacterial taxa. Four hours of incubation was chosen as the time is sufficient to look for responses in 16S rRNA levels despite the known low turnover rate of this molecule [65,66,67]. Exposure to these flavonols from food consumption in vivo would be over shorter intervals. However, there is evidence that polyphenols are sticky. Their consumption can be followed by long time intervals of availability to constituents of the oral biofilm based on their adherence to biofilms or bacteria themselves [45,68]. The flavonols tested can occur at high levels in many plant-based foods (Table 1), though levels tested here may not be achievable typically during food consumption. It is important to note that these are purified flavonols. Normally through eating one would be exposed to groups of polyphenols at cumulatively higher levels, which may work together. Finally, there is much discussion about using gums and lozenges for delivery of antibacterials, which could result in long-term exposure and potentially inhibition of activity of the same disease-associated bacteria identified in the shed biofilm assay. An important consideration is that as oral biofilms change with oral disease, susceptibility to flavonols may change.

## 5. Conclusions

In conclusion, the three flavonols tested largely affect the same bacterial taxa but at different levels. The other finding is that for the examples of atherosclerotic disease and oral disease, disease-associated bacteria are more frequently affected than ones associated with good health—it remains to be seen if these ex vivo findings can be translated to those seen in vivo and at what levels of flavonol intake.

## Figures and Tables

**Figure 1:**
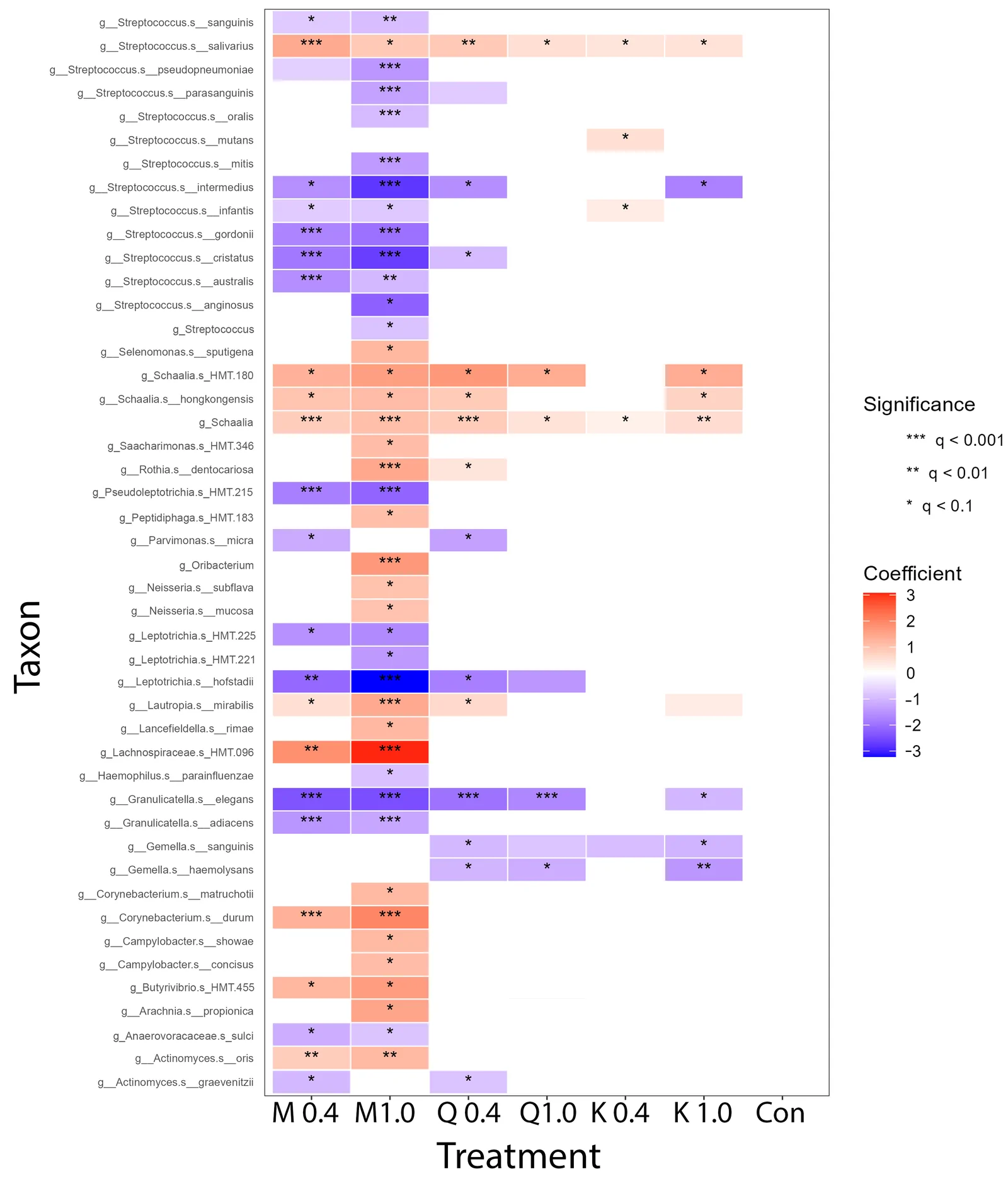
An examination of 16S rRNA levels 4 h after exposure to the various flavonols revealed taxa that showed inhibition or lack of inhibition versus mock exposed controls. Significant differences at * q < 0.1, ** q < 0.01, *** q < 0.001 are indicated. Color and intensity indicate effect size and whether positive or negative. Myricetin (M), kaempferol (K) and quercetin (Q) at 400 μM and 1 mM. Two vehicle controls assays were done, with one used as baseline in the paired analysis and the other analyzed as a negative control (Con).

**Figure 2:**
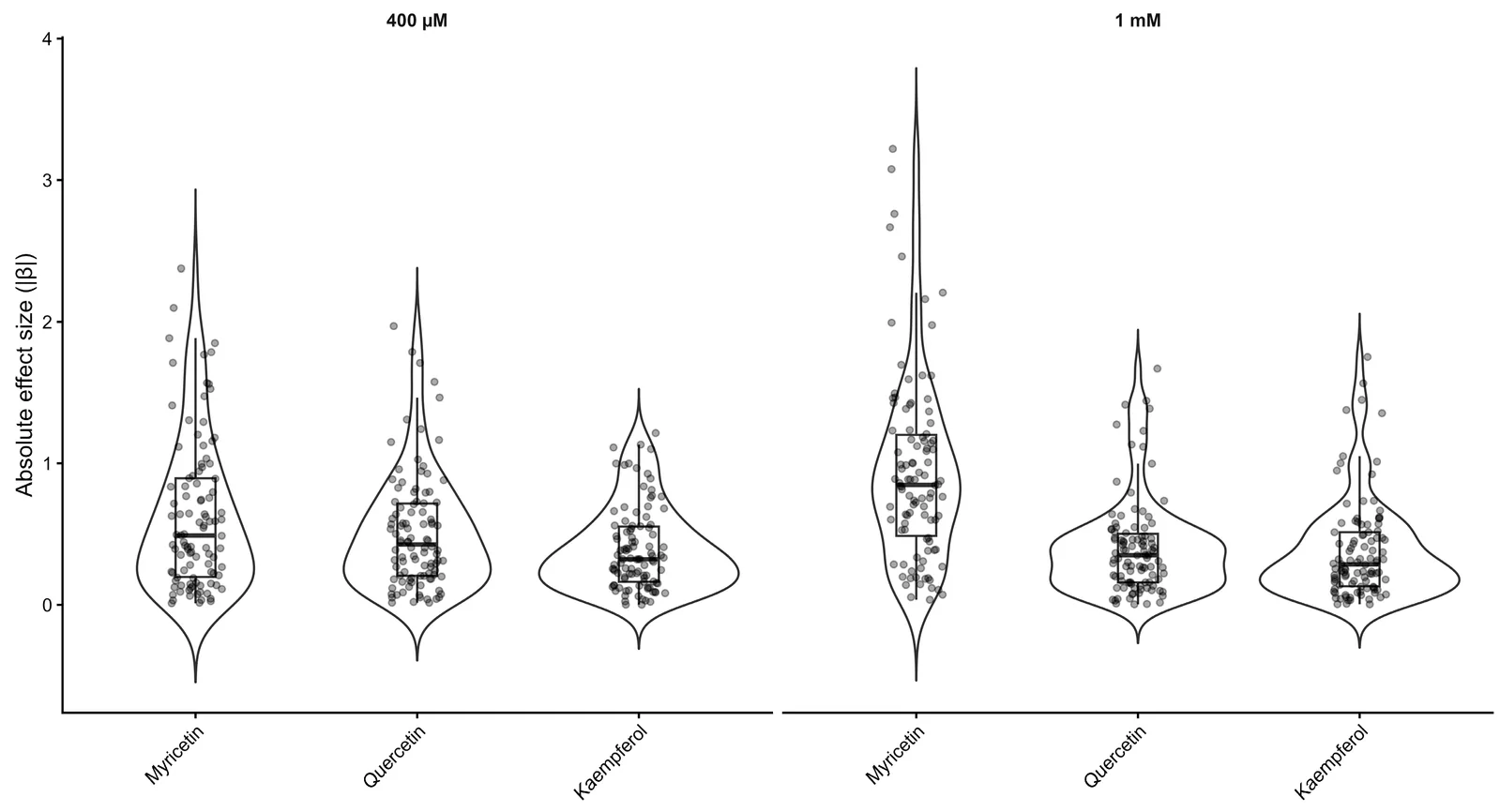
Absolute effect size comparison across the three flavonols tested at the 400 μM and 1 mM concentrations for the same 102 taxa 16S rRNA levels. Each point represents a taxon with boxplots showing the absolute effect size distribution, with myricetin having the largest mean and median effect size at 400 μM and 1 mM. A Friedman rank-sum test at 400 μM (χ^2^ = 7.31, *df* = 2, *p*-val = 0.0258) and 1 mM (χ^2^ = 68.02, *df* = 2, *p*-val = 1.70 × 10^−15^) showed significant differences in effect size magnitudes among the three flavonols.

**Figure 3:**
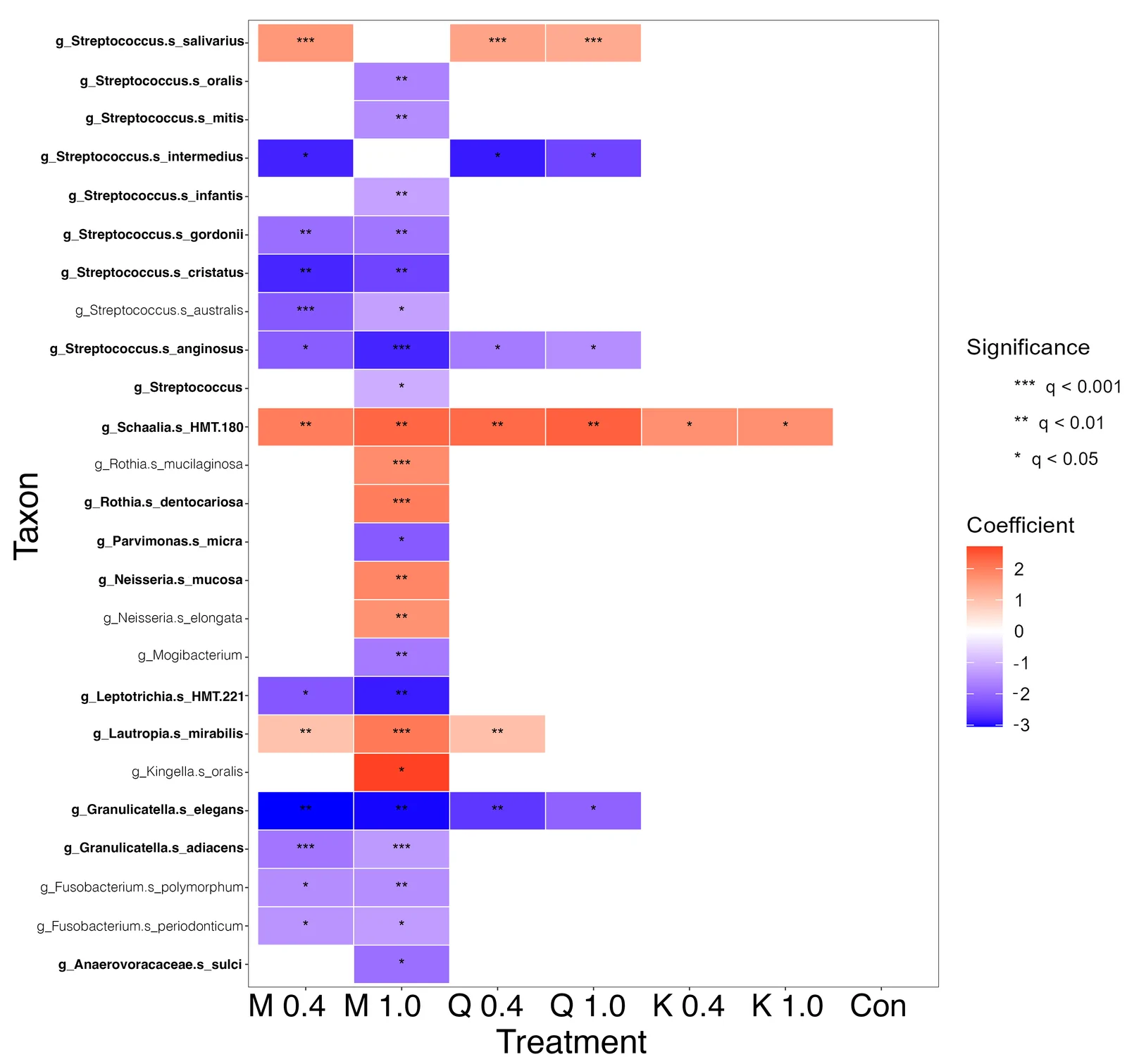
An examination of 16S rRNA levels 4 h after exposure to the various flavonols to shed biofilm from six additional donors revealed taxa that showed inhibition or lack of inhibition versus mock exposed controls. Significant differences at * q < 0.05, ** q < 0.01, *** q < 0.001 are indicated. Color and intensity indicate effect size and whether positive or negative.

**Figure 4:**
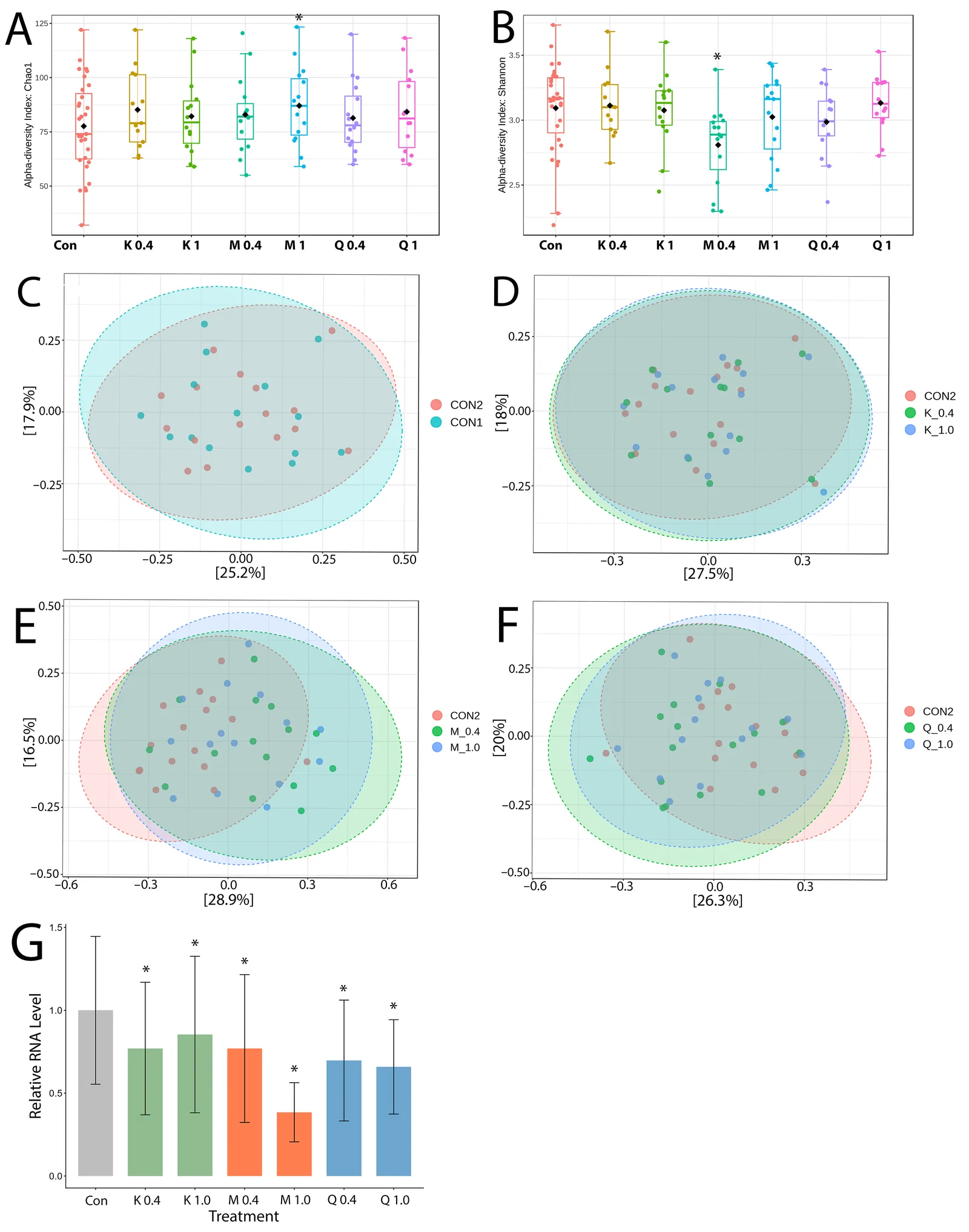
(**A**) Chao1 analysis comparison of taxa richness of the RNAs from the shed biofilm incubated with the three flavonols for all donors. Differences in 16S rRNA levels versus the control mock incubations are shown based on paired *t*-test. Significant differences in alpha diversity (FDR < 0.05) are marked by an asterisk. (**B**) Shannon analysis compared taxa diversity and richness of the RNAs from the biofilm incubated with the three flavonols. Significant differences in alpha diversity (FDR < 0.05) are marked by an asterisk. (**C**) Negative control: Beta diversity based on Bray–Curtis distances, Control1 versus Control2 (both vehicle only). PERMANOVA F-value 0.101 R-squared  =  0.0034 FDR  <  1.0. (**D**) Beta diversity based on Bray–Curtis distances, kaempferol versus the control. At 400 μM, PERMANOVA F-value was 0.262, and R-squared  =  0.0096 FDR  <  1.0 and at 1 mM PERMANOVA F-value was 0.465, and R-squared  =  0.163, FDR  <  1.0 (**E**) Beta diversity based on Bray–Curtis distances, myricetin versus the control. At 400 μM, PERMANOVA F-value 4.56, and R-squared  =  0.136, FDR  <  0.003 and at 1 mM PERMANOVA F-value 4.20, R-squared  =  0.127, FDR  <  0.003 (**F**) Beta diversity based on Bray–Curtis distances, quercetin versus the control. At 400 μM, PERMANOVA F-value 2.44, and R-squared  =  0.075, FDR  <  0.057 and 1 mM PERMANOVA F-value 1.16, R-squared  =  0.398, FDR  <  0.474. (**G**) Total RNA levels in the shed biofilm samples after exposure to the three flavonols at 400 μM and 1 mM for 4 h. RNA concentration of control was on average 80 ng/μL in 100 μL for each sample. All levels were lower than the control, incubated without polyphenol at FDR of <0.0003, with significant differences (FDR < 0.05) marked by an asterisk. PERMDISP verification showed homogeneity of multivariate dispersion across groups.

**Figure 5:**
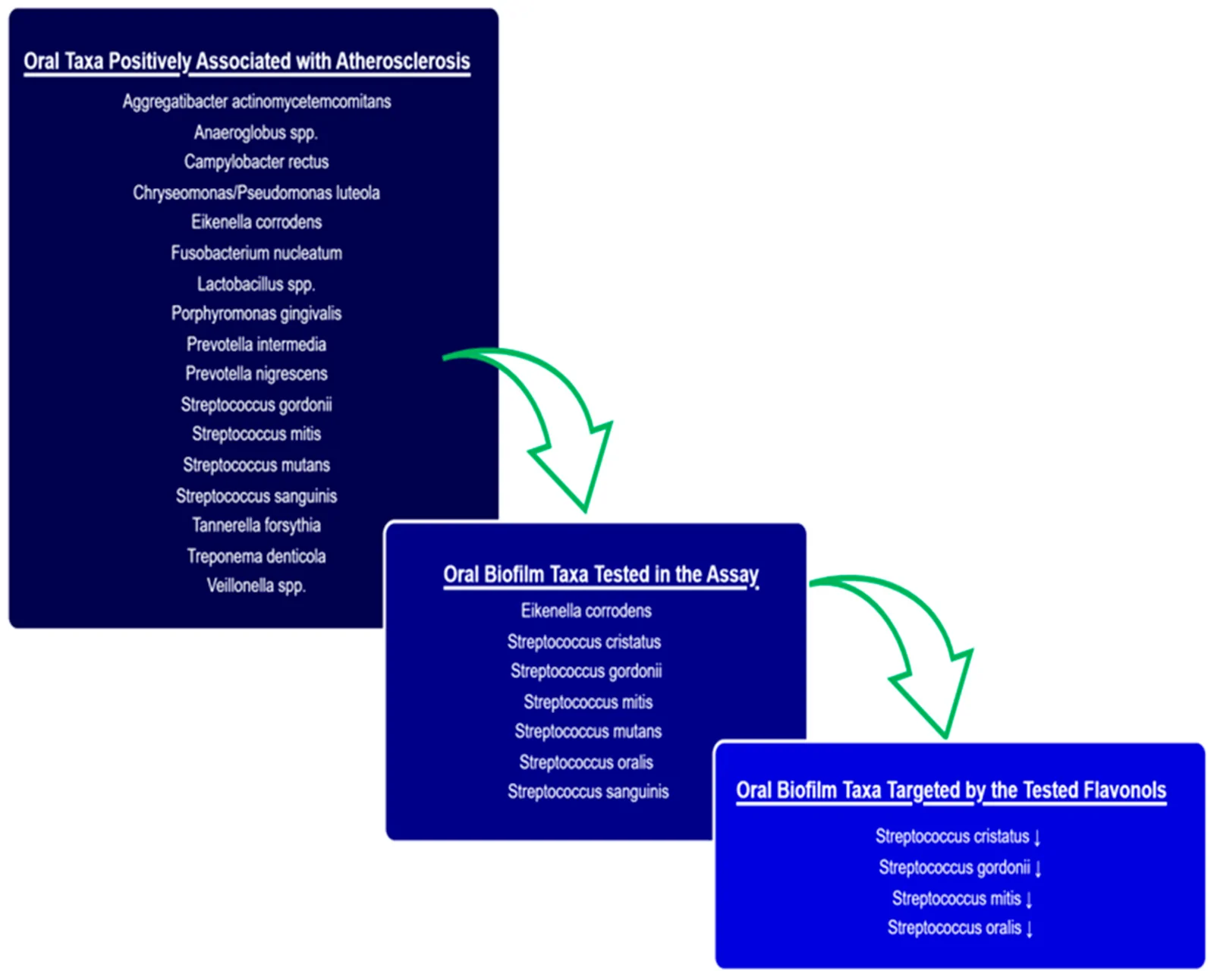
Of the oral bacteria associated with atherosclerosis [47,48,49,50,51] that were present in sufficient donors to allow testing, four were inhibited on the rRNA level by flavonols in the shed biofilm assay in both sets of tests, as indicated by a downward arrow.

**Table 1:** Table of the concentrations of three flavonols—myricetin, quercetin, and kaempferol—in fruits, vegetables, and beverages based on the 2018 USDA Database for Flavonoid Content (Release 3.3). Data as presented are mg/100 g, fresh weight, which is an edible portion.

	Flavonol		
	Myricetin	Quercetin	Kaempferol
Fruits			
Cranberries (raw)	7.63	16.64	0.09
Blackberries (raw)	0.67	3.58	0.27
Blueberries, cultivated (raw)	1.30	7.67	1.66
Sweet cherries (raw)	0.05	2.29	0.24
Apricots (raw)	--	1.63	0.63
Black grapes	0.22	2.08	0.09
Oranges (raw)	0.15	0.45	0.13
Strawberries (raw)	0.04	1.11	0.50
Vegetables			
Broccoli (raw)	0.06	3.26	7.84
Swiss chard, redleaf (raw)	2.20	7.50	9.20
Garlic (raw)	1.61	1.74	0.26
Wild ginger	--	--	33.60
Kale (raw)	--	22.58	26.80
Iceberg lettuce (raw)	0.06	0.15	1.42
Red onions (raw)	2.16	39.21	0.70
Fresh Parsley	14.84	0.28	1.49
Red ripe tomatoes (raw)	0.13	0.58	0.09
Beverages			
Green tea, brewed (decaffeinated)	1.00	2.77	1.00
Black tea, brewed (decaffeinated)	0.89	2.74	0.88
Red wine	0.42	1.04	0.09

## Data Availability

Sequence data in the form of 16S rRNA fastq files are available through the Sequence Read Archive with primary accession code: SUB16475303. (Submission in progress).

## Supplementary Materials

The following supporting information can be downloaded at: https://www.mdpi.com/article/10.3390/nu18193123/s1. Table S1. (A) Shared bacterial taxa that show differential relative levels of 16S rRNA from the group of 10 human donors and from an independent group of 6 donors. Effect sizes and q-values for the shared taxa of the 10-sample group at FDR < 0.1 after exposure of shed biofilm to the 3 flavonols versus the controls are displayed. (B) Shared bacterial taxa that show differential relative levels of 16S rRNA from the group of 10 human donors and from an independent group of 6 donors. Effect sizes and q-values for the shared taxa of the 6-sample group at FDR < 0.05 after exposure of shed biofilm to the 3 flavonols versus the controls are displayed.

## Abbreviations

The following abbreviations are used in this manuscript:

MIC	Minimal inhibitory concentration
MBC	Minimal bactericidal concentration
FDR	False Discovery Rate
q	False Discovery Rate
