# Supplementary material for: Targeting of Oral Bacterial Taxa Linked to Disease by Flavonols
Source: Nutrients. 2026 Sep 23;18(19):3123. doi: 10.3390/nu18193123 (PMC13667267; doi:10.3390/nu18193123)
Supplement: Supplementary file 1 [file nutrients-18-03123-s001.zip › nutrients-4554765-supplementary.pdf]

Targeting of oral bacterial taxa linked to disease by flavonols

Michaela Tamamidis[1], Anthony Lopez[1], Wei Li[2], S.J Green[3], Christine Wu[2], and Guy R. Adami[1,*]

[1] Department of Oral Medicine and Diagnostic Sciences, College of Dentistry, University of Illinois Chicago, 801 S. Paulina St., Chicago, IL, USA
[2] Department of Pediatric Dentistry, College of Dentistry, University of Illinois Chicago, 801 S. Paulina St., Chicago, IL, USA
[3] Genomics and Microbiome Core Facility, Rush University, Chicago, IL USA
* Correspondence: gadami@uic.edu

Supplementary Table S1A. Shared bacterial taxa that show differential relative levels of 16S rRNA from the group of 10 human donors and from an independent group of 6 donors. Effect sizes and q-values for the shared taxa of the 10-sample group at FDR < 0.1 after exposure of shed biofilm to the 3 flavonols versus the controls are displayed.

	M 0.4	M 1.0	Q 0.4	Q 1.0	K 0.4	K 1.0
**Streptococcus salivarius**	1.41<br>q = 6.05E-07	0.88<br>q = 2.27E-02	0.89<br>q = 1.07E-03	0.54<br>q = 4.27E-02	0.44<br>q = 4.12E-02	0.47<br>q = 2.98E-02
**Streptococcus oralis**	-	-0.94<br>q = 4.45E-04	-	-	-	-
**Streptococcus mitis**	-	-1.38<br>q = 5.48E-05	-	-	-	-
**Streptococcus intermedius**	-1.56<br>q = 7.83E-02	-2.76<br>q = 2.48E-02	-1.58<br>q = 8.03E-02	-	-	-1.75<br>q = 2.57E-02
**Streptococcus infantis**	-0.72<br>q = 4.39E-02	-0.75<br>q = 1.15E-02	-	-	0.32<br>q = 3.12E-02	-
**Streptococcus gordonii**	-1.71<br>q = 4.47E-04	-1.99<br>2.14E-05	-	-	-	-
**Streptococcus cristatus**	-1.88<br>q = 4.80E-07	-2.67<br>q = 3.88E-12	-0.96<br>q = 1.23E-02	-	-	-
**Streptococcus anginosus**	-	-2.21<br>q = 2.27E-02	-	-	-	-
**Streptococcus_**	-	-0.84<br>q = 1.15E-02	-	-	-	-
**Schaalia HMT.180**	1.29<br>q = 6.33E-02	1.59<br>q = 4.34E-02	1.71<br>q = 1.23E-02	1.39<br>q = 4.34E-02	-	1.38<br>q = 3.05E-02
**Rothia dentocariosa**	-	1.45<br>q = 3.75E-06	0.45<br>q = 8.26E-02	-	-	-
**Parvimonas micra**	-1.16<br>q = 6.55E-02	-	-1.31<br>q = 2.77E-02	-	-	-
**Neisseria mucosa**	-	1.00<br>q = 5.73E-02	-	-	-	-
**Leptotrichia HMT. 221**	-	-1.41<br>q = 7.57E-02	-	-	-	-
**Lautropia mirabilis**	0.54<br>q = 4.63E-02	1.43<br>q = 2.54E-05	0.64<br>q = 2.56E-02	-	-	-
**Granulicatella elegans**	-2.38<br>q = 6.05E-07	-2.46<br>q = 5.72E-07	-1.97<br>q = 9.98E-06	-1.67<br>q = 2.35E-04	-	-1.01<br>q = 8.24E-02
**Granulicatella adiacens**	-1.47<br>q = 1.71E-06	-1.24<br>q = 3.43E-05	-	-	-	-
**Anaerovoracaceae sulci**	-1.12<br>q = 2.27E-02	-0.82<br>q = 8.57E-02	-	-	-	-

Supplementary Table S1B. Shared bacterial taxa that show differential relative levels of 16S rRNA from the group of 10 human donors and from an independent group of 6 donors. Effect sizes and q-values for the shared taxa of the 6-sample group at FDR < 0.05 after exposure of shed biofilm to the 3 flavonols versus the controls are displayed.

	M 0.4	M 1.0	Q 0.4	Q 1.0	K 0.4	K 1.0
**Streptococcus salivarius**	1.63<br>q = 1.78E-05	-	1.44<br>q = 4.79E-05	1.34<br>q = 1.81E-04	-	-
**Streptococcus oralis**	-	-1.64<br>q = 2.98E-03	-	-	-	-
**Streptococcus mitis**	-	-1.50<br>q = 3.76E-03	-	-	-	-
**Streptococcus intermedius**	-2.82<br>q = 2.52E-02	-	-2.89<br>q = 1.67E-02	-2.46<br>q = 1.92E-02	-	-
**Streptococcus infantis**	-	-1.23<br>q = 1.88E-03	-	-	-	-
**Streptococcus gordonii**	-1.92<br>q = 5.97E-03	-1.81<br>q = 2.85E-03	-	-	-	-
**Streptococcus cristatus**	-2.80<br>q = 3.05E-03	-2.45<br>q = 4.92E-03	-	-	-	-
**Streptococcus anginosus**	-2.14<br>q = 1.29E-02	-2.81<br>q = 7.43E-04	-1.75<br>q = 2.23E-02	-1.49<br>q = 3.56E-02	-	-
**Streptococcus_**	-	-1.06<br>q = 2.35E-02	-	-	-	-
**Schaalia HMT.180**	2.00<br>q = 5.30E-03	2.31<br>q = 8.44E-03	2.27<br>q = 2.63E-03	2.38<br>q = 2.39E-03	1.74<br>q = 2.42E-02	1.73<br>q = 4.35E-02
**Rothia dentocariosa**	-	2.00<br>q = 9.16E-04	-	-	-	-
**Parvimonas micra**	-	-2.18<br>q = 1.14E-02	-	-	-	-
**Neisseria mucosa**	-	1.89<br>q = 9.13E-03	-	-	-	-
**Leptotrichia HMT. 221**	-2.21<br>q = 2.65E-02	-2.88<br>q = 5.24E-03	-	-	-	-
**Lautropia mirabilis**	0.97<br>q = 9.95E-03	2.06<br>q = 1.10E-04	1.01<br>q = 9.65E-03	-	-	-
**Granulicatella elegans**	-3.05<br>q = 3.05E-03	-3.01<br>q = 1.88E-03	-2.61<br>q = 9.04E-03	-2.07<br>q = 3.60E-02	-	-
**Granulicatella adiacens**	-1.80<br>q = 2.06E-05	-1.32<br>q = 6.18E-04	-	-	-	-
**Anaerovoracaceae sulci**	-	-1.90<br>q = 1.67E-02	-	-	-	-
